# Research and Prospects of Digital Twin-Based Fault Diagnosis of Electric Machines

**DOI:** 10.3390/s25082625

**Published:** 2025-04-21

**Authors:** Jiaqi Hu, Han Xiao, Zhihao Ye, Ningzhao Luo, Minhao Zhou

**Affiliations:** School of Electrical Engineering, Naval University of Engineering, Wuhan 430033, China; yxyx928@126.com (Z.Y.); ningzhaoluo@gmail.com (N.L.); m23385812@nue.edu.cn (M.Z.)

**Keywords:** digital twin, motor, fault diagnosis, data driven

## Abstract

This paper focuses on the application of digital twins in the field of electric motor fault diagnosis. Firstly, it explains the origin, concept, key technology and application areas of digital twins, compares and analyzes the advantages and disadvantages of digital twin technology and traditional methods in the application of electric motor fault diagnosis, discusses in depth the key technology of digital twins in electric motor fault diagnosis, including data acquisition and processing, digital modeling, data analysis and mining, visualization technology, etc., and enumerates digital twin application examples in the fields of induction motors, permanent magnet synchronous motors, wind turbines and other motor fields. A concept of multi-phase synchronous generator fault diagnosis based on digital twins is given, and challenges and future development directions are discussed.

## 1. Introduction

As a core device that converts electrical energy into mechanical energy, the motor plays an indispensable role in industrial production, transportation, daily life and many other fields [1]. However, due to their working environment often being more complex, and their need to run for a long time, motors are very prone to various types of failure. These faults not only can interfere with the normal operation of equipment, resulting in production interruption and efficiency reduction, but also may cause safety accidents, resulting in serious economic losses [2]. Therefore, accurate and timely diagnosis of motor faults has become a key task to ensure the reliable operation of motors [3].

In recent years, with the rapid development of information technology, motor fault diagnosis technology is also constantly being revolutionized [4]. From the traditional method based on sensor monitoring and simple data analysis, the methods for fault diagnosis have gradually developed into intelligent diagnostic means using advanced technologies such as data mining and machine learning [5]. Among them, digital twin technology, as a cutting-edge digitalization technology, has become a research hotspot in this field by virtue of its unique features of virtual–real mapping, its data-driven nature, model fusion, real-time interaction and full lifecycle coverage, which bring brand new ideas and methods for motor fault diagnosis [6]. The purpose of this paper is to provide a systematic review of the application of digital twin technology in electric motor fault diagnosis, sort out its development status, key technologies and application examples, give a conception of multi-phase synchronous generator fault diagnosis based on digital twins, and discuss challenges and future development directions.

## 2. Current Status of Research on Digital Twins

### 2.1. Origins and Development of Digital Twins

#### 2.1.1. Origins of the Digital Twin

**Figure 1:**
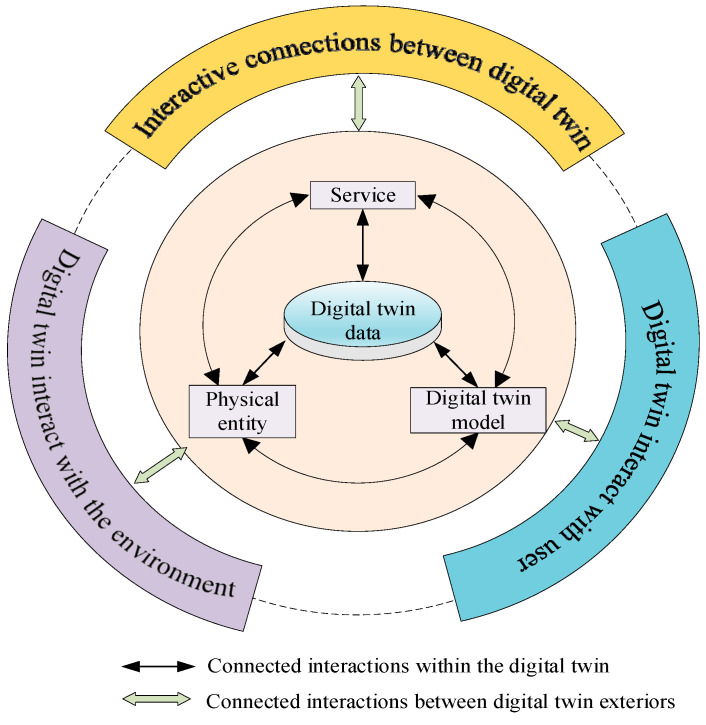
Five-dimensional model of digital twin.

**Figure 2:**
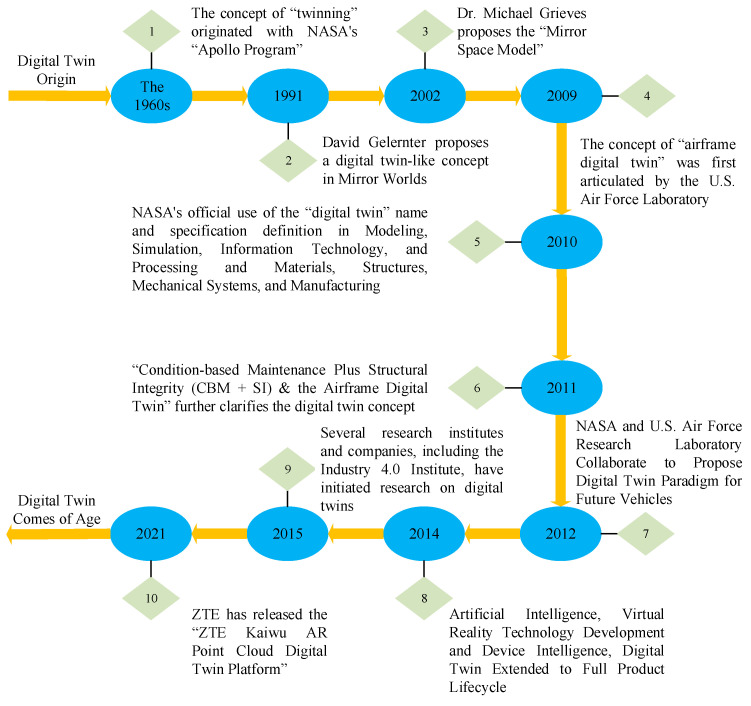
Evolution of digital twin technology.

The concept of digital twin can be traced back to 2002, when Professor Grieves of the University of Michigan first proposed the concept of “Mirrored Space Model” (Mirrored Space Model) in his Product Lifecycle Management (PLM) course, which laid the foundation for the birth of digital twin [7]. The concept of Mirrored Space Model was first introduced by Prof. Grieves in his Product Lifecycle Management (PLM) course, which laid the foundation for the birth of digital twins. Subsequently, Prof. Grieves further elaborated on the digital twin in his 2011 book and defined it as a virtual information structure used to describe products from the micro-atomic level to the macro-geometric level [8]. Thereafter, the National Aeronautics and Space Administration (NASA) formally defined digital twins as a multi-physics field, consisting in multi-scale, probabilistic, ultra-fidelity simulation in 2012, fully utilizing a physical model, sensor data and historical operational data to reflect the state of corresponding physical entities in real time [9]. Prof. Tao Fei of Beijing University of Aeronautics and Astronautics previously conducted deep theoretical research and explored application practices for digital twins, mainly including digital twin workshops, digital twin-based design and manufacturing, and a five-dimensional digital twin model [10]. Figure 1 shows the five-dimensional model of digital twins proposed by Prof. Tao Fei, and Figure 2 shows the development history of digital twin technology.

#### 2.1.2. The Concept of a Digital Twin

A digital twin is a virtual digital model that reflects and predicts the state and behavior of a physical entity in real time. Its core concepts can be elaborated in the following aspects:

(1) Virtual–reality mapping. The digital twin collects and transmits real-time data of the physical entity to the virtual space through sensors and other technical means, and constructs a dynamic mapping relationship between the physical entity and the virtual model, which enables the virtual model to truly reflect the real-time situation of the physical entity [10].
(2) Data-driven. The construction and operation of digital twins rely on massive amounts of real-time data. These data come from the physical entity’s sensors, historical operation records and the external environment. Through the analysis and processing of these data, the digital twin can continuously optimize its own model parameters, thus improving the simulation accuracy and prediction ability of the physical entity [11].
(3) Model fusion. Digital twins usually consist of multiple models at different levels and in different domains, including geometric, physical, behavioral and rule models. These models are interrelated and fused together to form a complete digital twin system. Through model fusion, digital twins are able to comprehensively describe and analyze physical entities from multiple perspectives, providing powerful support for modeling and simulation of complex systems [12].
(4) Real-time interaction. There is a real-time two-way interaction between the digital twin and the physical entity. On the one hand, the digital twin can be dynamically updated and optimized according to the real-time data of the physical entity; on the other hand, the simulation results and prediction information of the digital twin can be fed back to the physical entity to provide a basis for its operation control and decision-making [13].
(5) Full lifecycle coverage. The digital twin runs through the entire lifecycle of a physical entity, from product design and manufacturing to operation and maintenance and end-of-life recycling. At different stages, digital twins can play different roles, such as virtual validation and optimization at the design stage, guidance of the production process at the manufacturing stage, and condition monitoring and fault prediction at the operation stage [14].

As an emerging digital technology, the origin and development history of digital twins reflects the trend of deep integration of information technology and physical systems. From the perspective of the core concept of digital twins, the features of virtual–reality mapping, data-drivenness, model fusion, real-time interaction, and full lifecycle coverage together constitute the unique advantages of digital twins.

### 2.2. Key Technologies and Applications of Digital Twins

#### 2.2.1. Digital Twin Key Technologies

(1) Modeling technology. Digital twin modeling is the foundation of the technology, and the characteristics of multiple aspects of physical entities need to be considered to construct an accurate model. Multi-physics field modeling integrates the multi-physics field characteristics of physical entities; e.g., in the aerospace field, hydrodynamics, structural mechanics, and other multi-physics fields are coupled in the modeling of aircraft to accurately simulate flight states [10]. Multi-scale modeling covers different scale information from microscopic to macroscopic levels, and in the manufacturing industry, the atomic structure of microscopic parts and components is covered for overall performance modeling of the macroscopic product, which comprehensively reflects the product characteristics [11]. Data-driven modeling is based on a large number of historical data mining laws to establish models, such as in power equipment fault diagnosis, based on equipment operation data to establish fault prediction models to improve diagnostic accuracy [12].
(2) Data processing technology. Data collection collects physical entity multi-source heterogeneous data through sensors, and collects equipment operation parameters, environmental data, etc., in industrial production [13]. Data cleaning removes erroneous and redundant data to improve data quality. Feature extraction refines key features from raw data to provide support for subsequent analysis and decision-making. Data fusion integrates multi-source data, such as the fusion of vehicle sensors, road conditions and other data in intelligent transportation systems to improve the reliability of system decision-making [14].
(3) Communication technology. Communication technology realizes real-time interactions between physical entities and virtual model data. Fifth-generation technology has high speed, low latency and large connection characteristics, which meet the demand for real-time transmission of large amounts of data from digital twins and facilitate efficient communication between devices and models on the industrial Internet [15]. Industrial Ethernet is widely used in factory automation scenarios for its high reliability and real-time performance, guaranteeing stable transmission of equipment data [16]. Wireless sensor networks are suitable for data acquisition and transmission in complex environments, such as in building structural health monitoring, where wireless sensors are deployed to obtain structural status data [17].
(4) Intelligent decision-making technology. Intelligent decision-making techniques are based on digital twin models and data to achieve optimal decision-making. Machine learning algorithms such as neural networks, support vector machines, etc., analyze and train on the digital twin data to achieve fault diagnosis, playing an important role in wind power equipment fault diagnosis [18]. Deep learning in image recognition, speech processing and other aspects performs remarkably and can help digital twins deal with complex data, improving the level of decision-making intelligence, such as in the field of intelligent security for image analysis to identify abnormal behavior [19]. Expert systems reason and make decisions based on the knowledge and experience of domain experts, providing professional decision support for digital twins—e.g., assisting doctors in judging medical conditions in medical diagnostic assistance systems [20].

#### 2.2.2. Digital Twin Application Areas

**Figure 3:**
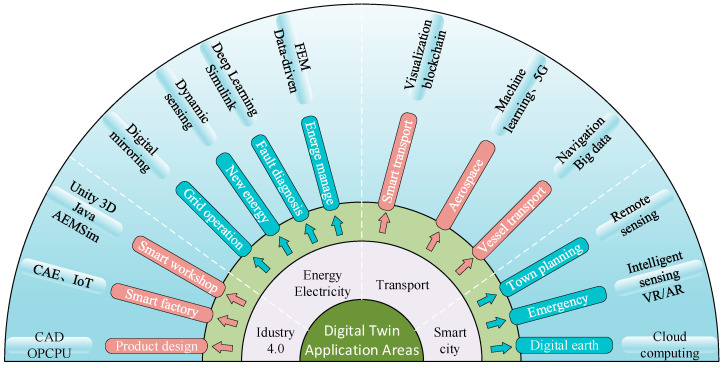
Application areas of digital twin technology.

Since the concept of digital twin was proposed in 2002; with the development of information technology, it has gradually moved from theory to practical application and become a hotspot of research in multiple fields. Figure 3 gives the main application areas of digital twin technology.

(1) Smart manufacturing field. In the product design stage, virtual models are constructed for simulation and optimization. Design defects are found in advance, and the R&D cycle is shortened; e.g., digital twins are used to optimize the design of body structures in automobile manufacturing [21]. In the production process, digital twins enable real-time monitoring of equipment operating status and prediction of failures and timely maintenance to improve production efficiency and product quality; e.g., factories through digital twins can monitor the operating parameters of equipment to predict failures [22]. In the after-sales stage of products, product usage data can be analyzed based on digital twins to improve product performance and service; e.g., home appliance companies optimize product functionality by analyzing user usage data [23].
(2) Energy and power field. In power generation, generator sets are modeled to monitor the operating status in real time, predict failures and performance degradation and optimize power generation efficiency—e.g., monitoring boiler operating parameters to predict failures in thermal power generation [24]. In transmission, digital twin models of transmission lines are constructed to monitor line status, assess fault risk and improve transmission reliability; e.g., digital twins are used to monitor transmission line ice cover [25]. Distribution links, as supported by digital twins, help realize digital management of distribution networks and optimize power distribution—e.g., analyzing distribution network load distribution to optimize power supply schemes through digital twins [26].
(3) Transportation field. In intelligent transportation systems, data on vehicles, roads and traffic flow are fused to optimize traffic signal control and route planning to alleviate traffic congestion [27]. In the field of aerospace, the modeling of aircraft and engines to monitor performance and health and to ensure flight safety uses digital twins—e.g., the use of digital twins by aircraft manufacturers to monitor engine operation status [28]. In ship transportation, real-time monitoring of ship navigation status uses digital twins, optimizing route planning and improving transportation efficiency and safety—e.g., analyzing ship navigation data to optimize routes through digital twins [29].
(4) Smart city field. In urban planning, the construction of urban digital models to simulate the development of a city provides a basis for planning decisions—e.g., simulating the development of the city to assess the feasibility of planned programs [30]. In urban management, digital twins help realize real-time monitoring and intelligent management of urban infrastructure, environment, transportation, etc. One example of an application is monitoring urban environmental data to realize intelligent environmental protection [31]. In emergency management, digital twins are used to simulate disaster scenarios, develop emergency plans and improve emergency response capabilities [32].

## 3. Current Research Status of Motor Fault Diagnosis Technology

**Figure 4:**
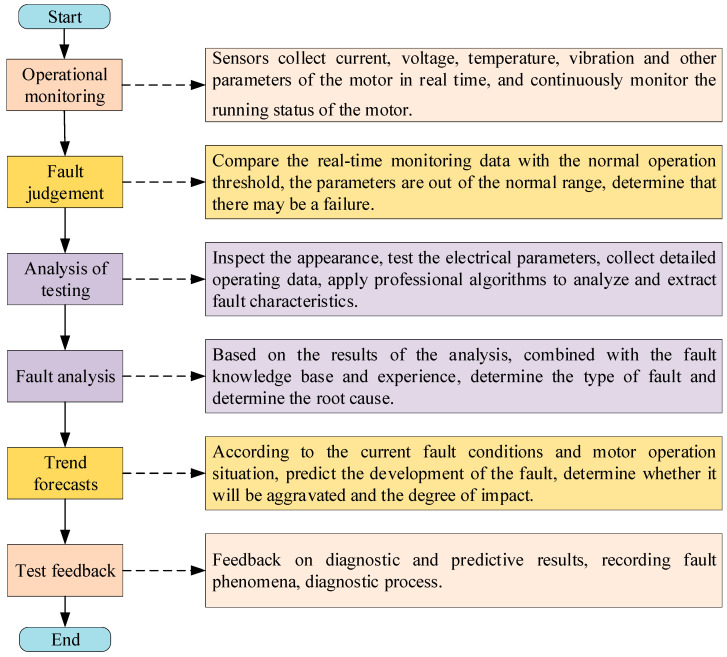
Conventional motor fault diagnosis process.

As an important piece of equipment for electromechanical conversion, motors play a key role in industrial production, transportation, daily life and many other fields. However, due to the complex working environment, long-term operation and other factors, motors are prone to various failures. Accurately identifying the types of motor faults, deeply exploring the causes of faults and applying effective diagnostic methods to find and solve problems in a timely manner have become core tasks to ensure the reliable operation of motors. Figure 4 gives the traditional motor fault diagnosis process.

### 3.1. Types and Causes of Common Motor Faults

#### 3.1.1. Electrical Faults

(1) Stator winding faults: Stator winding faults are the more common types of electrical faults in motors, including phase-to-phase short circuits, turn-to-turn short circuits and grounded short circuits. Reference [33] states that about 32% of the damage to induction motors is caused by short circuit faults due to insulation failure. Insulation aging is one of the main causes of stator winding short circuits. During long-term operation, the electromagnetic stress, thermal stress and environmental factors inside the motor will gradually degrade the performance of the insulating material, ultimately reducing the insulating ability. In addition, mechanical damage, moisture, etc., may also trigger stator winding short circuiting. During the manufacturing, installation or operation of a motor, if it is impacted by external forces, the winding insulation may be damaged; and when motors are operated in a humid environment, moisture intrusion will reduce the insulation performance and increase the probability of short circuit failure [34]. Reference [35] states that 80% of stator electrical faults are caused by weak turn-to-turn insulation. After an ITSC fault occurs in a nine-phase PMSM, the stator windings will form a short circuit loop, which generates a large short circuit current under the action of the magnetic field, causing severe damage to the motor. Reference [36] points out that the insulation aging caused by long-term operation of asynchronous motors, overload caused by abnormal loads and poor contact caused by wear and tear of components will make direct contact between the windings and cause short circuiting of the rotor windings.
(2) Rotor failure: Rotor failure mainly includes rotor broken bars and rotor imbalance. The broken rotor strip will cause motor output torque reduction, abnormal vibration and noise during operation. As described in [37], when the motor is frequently started, braked or overloaded, the rotor guide bar will be subjected to significant mechanical stress and electromagnetic forces, which can easily lead to the fracture of the guide bar in the long term. In addition, the poor quality of rotor materials and the presence of internal defects also increase the risk of rotor bar breakage. Rotor imbalance is usually caused by rotor manufacturing process errors, improper installation or localized wear during operation. An unbalanced rotor generates centrifugal force when rotating, resulting in increased vibration of the motor, which not only affects the service life of the motor, but also may cause damage to the surrounding equipment [38].
(3) Demagnetization failure: When the PMSM operates at high speeds or high loads, the temperature rises significantly, causing irreversible demagnetization of the permanent magnets. The demagnetization fault will degrade the performance of the motor, as evidenced by the change in the output characteristics of the motor under different load and speed conditions. Reference [39] investigated three demagnetization fault states of PMSM—normal, mild demagnetization (10% demagnetization) and severe demagnetization (25% demagnetization)—and pointed out that the temperature of the PMSM increases significantly during high-speed or high-load operation, which may result in irreversible demagnetization of the permanent magnets, and then degrade the performance of the motor. Reference [40] points out that the operating conditions of PMSM are complex, and factors such as inrush currents generated by armature reactions, severe mechanical vibrations and excessive temperatures may lead to irreversible demagnetization of the permanent magnets.

#### 3.1.2. Mechanical Faults

(1) Bearing failure: Bearing failure occupies a high proportion of motor failures and is mainly manifested as bearing wear, fatigue spalling, poor lubrication and so on. According to [41], mechanical friction during long-term operation is an important cause of bearing wear, while insufficient lubrication, impurity intrusion and other factors will further aggravate the degree of wear. According to [42], when the bearing is poorly lubricated, the friction coefficient increases, which accelerates the wear of the bearing; if impurities enter into the bearing interior, it will destroy the normal operation of the bearing and trigger fatigue spalling. According to [43], inner ring failure is a common type of motor bearing failure, and during the operation of the motor, when the bore size of the inner ring is different, there will be defects such as breakage and holes in the inner ring. Reference [44] states that factors covering load variations, insufficient cooling, manufacturing defects, current problems, shaft clearance, transient voltage events and voltage imbalance can put additional stress on the internal components of the motor, accelerating the wear and aging of components and ultimately leading to the occurrence of bearing failures.
(2) Other mechanical failures: The motor may also experience mechanical problems such as brush wear and collector ring failure. During the operation of the motor, the brushes and the collector ring are in constant contact and friction, and if the contact pressure is uneven or the current density distribution is abnormal, it will lead to increased localized wear of the brushes [45]. Collector ring failures are usually manifested as surface oxidation, burns, etc., which are closely related to the electrical performance and environmental factors during motor operation [46]. In an environment of high humidity or the presence of corrosive gases, the surface of the collector ring is easy to oxidize, which affects its electrical conductivity; and when the motor is overloaded or short-circuited and suffers other abnormalities, the excessive current will cause the surface of the collector ring to be burned [47].

### 3.2. Motor Fault Diagnosis Methods and Applications

#### 3.2.1. Sensor-Based Diagnostic Methods

Sensor-based diagnostic methods collect parameters such as vibration, temperature and current during motor operation in real time by installing various types of sensors on the motor, and then determine the motor’s operating status. Reference [48] utilizes sensors to collect data such as the current, vibration and temperature of induction motors as a way to monitor the operating status of motors. Reference [49] proposes a CNN-based fault diagnosis system for induction motors, which uses vibration sensors to collect the vibration signals generated during the operation of the motor, digitize and store them, and finally input these data into a CNN model to determine whether the motor is in a normal, rotor fault, or bearing fault state. Reference [50] points out that large motor equipment is usually installed with vibration sensors and temperature sensors, and through real-time monitoring of these parameters, once abnormalities are found, timely measures can be taken to avoid further expansion of the fault and to protect the continuity of production.

#### 3.2.2. Diagnostic Methods Based on Data Analysis

(1) Time-domain analysis method: Time-domain analysis is to directly analyze the collected motor operation data in the time domain, such as observing the waveform changes of current and voltage signals. However, ref. [51] points out that time-domain analysis has limitations when facing complex faults, which makes it difficult to accurately extract fault characteristics, and for some minor faults, thee changes in the time-domain signals may not be obvious and can be easily ignored.
(2) Frequency-domain analysis method: Frequency-domain analysis involves converting the time-domain signal to the frequency domain by means of Fourier transform and other means to analyze the frequency components of the signal, so as to find out the fault characteristic frequency [52]. Through the frequency-domain analysis of the motor vibration signal, if it is found that the amplitude at a specific frequency increases abnormally, it can be judged that there may be rotor imbalance, a bearing failure and other problems. Reference [53] proposes a generalized diagnostic method based on stator current spectral features and motor speed using an SVM algorithm. The time-domain waveform of load current is obtained through an experiment, and the spectrum is obtained by fast Fourier transform, and the 30 Hz and 90 Hz frequency components are extracted as the SVM features, while the motor speed is used as an additional feature to improve the accuracy of rotor broken bar fault diagnosis [54].
(3) Data mining and machine learning methods: Reference [55] used a particle swarm optimization algorithm-optimized least squares support vector machine (PSO—LSSVM) approach to diagnose motor faults, which significantly improves the accuracy and efficiency of fault diagnosis by learning and training on a large amount of fault data. Literature [56] proposed an edge intelligence-based application deployment method for motor fault diagnosis, which achieves efficient and accurate fault diagnosis on resource-constrained edge devices by integrating multi-scale convolutional neural networks, long- and short-term memory networks and attention mechanisms, combined with knowledge distillation and model quantization techniques. Reference [57] constructs a CNN-based fault diagnosis system for induction motors. The CNN model analyzes the input data and then determines whether the motor is in a normal state, a rotor fault state or a bearing fault state. In view of the strong RF classification capability and CNN feature extraction capability, ref. [58] proposed to combine the two to form a CNN-RF model. The model first utilizes CNN to extract features from the fault data, and then RF diagnoses the fault type based on the extracted features. Experiments show that compared with other models, the CNN-RF model has a higher accuracy in motor fault diagnosis.

#### 3.2.3. Model-Based Diagnostic Methods

(1) Digital twin model: A digital twin model can comprehensively reflect the physical characteristics and operating state of a motor, providing a more accurate basis for fault diagnosis and having broad application prospects in the fields of wind power, aerospace and others requiring high reliability of the motor. Reference [59] proposed a wind turbine drive train fault diagnosis method based on a digital twin, through the establishment of a digital twin model of the motor, real-time simulation of the motor’s operating state and comparison and analysis with the actual operating data, so as to realize the diagnosis and prediction of motor faults. Reference [60] states that in wind farms, the use of digital twin models allows real-time monitoring and fault prediction of wind turbine motors, scheduling maintenance in advance, reducing operation and maintenance costs and improving power generation efficiency.
(2) Deep learning model: Deep learning models have powerful learning and adaptive capabilities, and can accurately recognize complex motor fault patterns [61,62]. The deep learning network-based asynchronous motor fault identification method proposed in the [63] first utilizes SAE to reduce noise and dimensionality of the fault dataset, and then uses CNN to automatically learn the deep features of the fault data. The method has strong adaptability and generalization ability to non-linear fault data, and comprehensively improves the accuracy, robustness and efficiency of asynchronous motor multi-fault diagnosis. Reference [64] proposes a prototype refinement method based on Deep Reinforcement Learning (DRL) for semi-supervised few-sample motor fault diagnosis to solve the problem of data scarcity in industrial scenarios, and experimentally verifies the effectiveness of the method in cross-category and cross-condition diagnosis tasks. In motor fault diagnosis, the neural network constructs a fault diagnosis model by learning the feature data of the motor under normal operation and various fault states. Literature [65] combines tools such as continuous wavelet transform, DFT, STFT, etc., and realizes automated diagnosis with the help of an adaptive neural network fuzzy inference system (ANFIS), which is able to detect the number of faulty turns and faulty phases.

#### 3.2.4. Comparative Analysis of Different Diagnostic Methods

In the field of motor fault diagnosis, different diagnostic methods have their own characteristics and application value. Table 1 shows a comparison of traditional motor fault diagnosis methods. In the actual application, the type of motor, operating environment, fault characteristics and diagnostic needs should be considered comprehensively, enabling the flexible selection or combination of multiple diagnostic methods to achieve complementary advantages, so as to enhance the reliability and effectiveness of motor fault diagnosis and ensure the safe and stable operation of motors.

#### 3.2.5. Areas of Application of Diagnostic Methods

(1) Industrial field: In industrial production, the normal operation of a motor, as a core component of mechanical equipment, is directly related to production efficiency and product quality. The various diagnostic methods mentioned above have been widely used in industrial motor fault diagnosis. Reference [66] points out that in the automated production line of a factory, through real-time monitoring of the motor’s operating data and the use of diagnostic methods based on sensors and data analysis, it is possible to discover hidden motor faults in a timely manner to ensure the stable operation of the production line. Reference [67] takes an elevator as an example, where the elevator had a permanent magnet synchronous motor as a key component; the reliability of its operating state is crucial. Using the fault diagnosis method for data analysis, combined with Kalman filtering, it is possible to discover minor faults in the motor over time and avoid further deterioration of the faults. In industrial scenarios using motors, ref. [68] constructs a network structure containing feature extractors, classifiers and unseen fault detectors, utilizing online samples and samples of each known category for adversarial training, for monitoring the operating status of motors, and for timely detection of faults and diagnosis.
(2) Energy field: In the energy industry, such as wind power and thermal power generation, the reliability of motors directly affects the production and supply of energy. Fault diagnosis of wind turbines based on digital twin and machine learning methods in [69] effectively guarantees the stable operation of wind power systems. Through real-time monitoring and fault diagnosis of motors, maintenance plans can be formulated in advance to reduce downtime and improve the reliability and economy of energy production [70]. In smart power plants, ref. [71] uses multi-sensor data acquisition and SDP image conversion to collect multi-source signals such as data on the current, electromagnetism and vibration of the motor in real time, having installed Hall current sensors, vibration sensors and high-frequency AC magnetic field probes on the motor. These signals are converted into SDP images and input into the MSF-SACapsNet model for analysis, which can accurately identify whether the motor is in a state of rotor imbalance, rotor misalignment, bearing failure, etc.
(3) Transportation: In the field of transportation, motors are widely used in electric vehicles, electric trains and other equipment. Accurate fault diagnosis is crucial to ensure the safety and smoothness of transportation. Diagnostic methods based on sensors and data analysis can monitor the running status of motors in real time, detect faults over time, avoid accidents during operation, and guarantee the safety of passengers and normal transportation [72]. In the CRH2 high-speed train traction system, in view of the non-linearity of the induction motor, ref. [73] proposes constructing a T-S fuzzy model to represent the operating state of the motor at different speeds with multiple linear submodels, and then combine these submodels into a global system model through the fuzzy affiliation function, so as to effectively deal with the non-linear problem of the motor.
(4) Other fields: Motor troubleshooting techniques are also used in smart homes and medical devices. In smart home systems, motors are used to drive various types of equipment. By monitoring and diagnosing the operating status of motors, faults can be detected in a timely manner, improving the reliability of home equipment and user experience [74]. In the field of medical devices, the use of advanced fault diagnosis methods can monitor the status of motors in some life-support equipment in real time to ensure the normal operation of the equipment, providing reliable protection for medical work [75]. In some high-end medical equipment, such as magnetic resonance imaging machines and intensive care monitors, the use of fault diagnosis technology for real-time monitoring of the motors in these devices can detect potential problems in real-time and avoid equipment failures that can cause harm to patients [76].

**Table 1:** Comparison of traditional fault diagnosis methods for motors.

Reference Signal	Diagnosis Method	Advantages	Limitations	Reference
Vibration	Bayesian algorithms, SVM, KNN	high diagnostic efficiency and generalization capability	Insufficient diagnostic capability for complex conditions	[44]
Vibration	CNN, GUI	High accuracy	\	[49]
Vibration	CNN, LSTM	High diagnostic accuracy and speed	Limited application scenarios	[56]
Vibration	DRL	Good small sample fault diagnosis	Large computational and experimental costs	[62]
Current	FFT, KNN, SVM	Effective extraction of key features for high diagnostic accuracy	Feature extraction relies on expert knowledge	[43]
Current	Cubic Fractional Order Model, Traceless Kalman Filter	High diagnostic accuracy for minor faults considering	\	[65]
Current	T-S fuzzy model, fault observer	Robust and sensitive, high accuracy	\	[70]
Flux	Search coils, harmonic magnetic fields	Low cost and fast	Simulation-based, not practical enough	[35]
Power Torque	Autoencoder and K-means	High accuracy without sensors	\	[39]
Magnetic Flux Density	FEM	Non-invasive diagnostics to reduce testing costs	\	[40]
Current RPM	FFT, SVM	High diagnostic accuracy for minor faults	No mention of simultaneous occurrence of multiple failures	[53]
Vibration Current	Model-based, signal processing, data-driven	Automatic fault recognition and extraction	Insufficient precision, low resolution	[57]
Vibration Current	CNN, LeakyRelu Function	High robustness and generalization	\	[61]
Current Torque	LabVIEW Motor Model, CNN-RF Model	High accuracy	\	[58]
Torque	FT, DWT	Utilization of mathematical tools, wide range of diagnostics	Poor low-speed diagnostics and high hardware costs	[63]
Acceleration	Knowledge distillation strategies for predicting score ordering	Automatic fault detection with high diagnostic accuracy	\	[66]
Vibration Current	Capsule network with self-attention mechanism	High diagnostic accuracy and robustness	High consumption of computing resources	[69]

## 4. Research on Key Technologies and Applications of Digital Twins in Motor Fault Diagnosis

**Figure 5:**
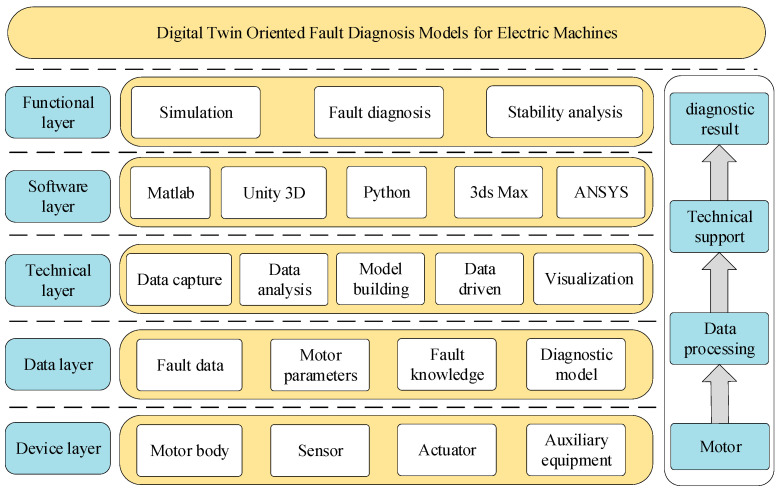
Digital twin-based motor fault diagnosis model architecture.

Motor fault diagnosis technology is constantly developing, gradually evolving from traditional sensor-based monitoring and simple data analysis to the use of data mining, machine learning and other advanced technologies, achieving promising results in motor fault diagnosis in various fields. In practical applications, the existing diagnostic techniques still face many challenges, while traditional methods often have difficulty comprehensively accessing operating state information of motors in real time, resulting in insufficient diagnostic accuracy for some complex faults and early faults. With the acceleration of industrial intelligence processes, motor systems will become more and more complex, resulting in higher requirements concerning the accuracy, timeliness and intelligence of fault diagnosis. Against this background, digital twin technology stands out with its unique advantages and opens up a new path for motor fault diagnosis. Figure 5 shows the architecture diagram of digital twin-based motor fault diagnosis.

**Figure 6:**
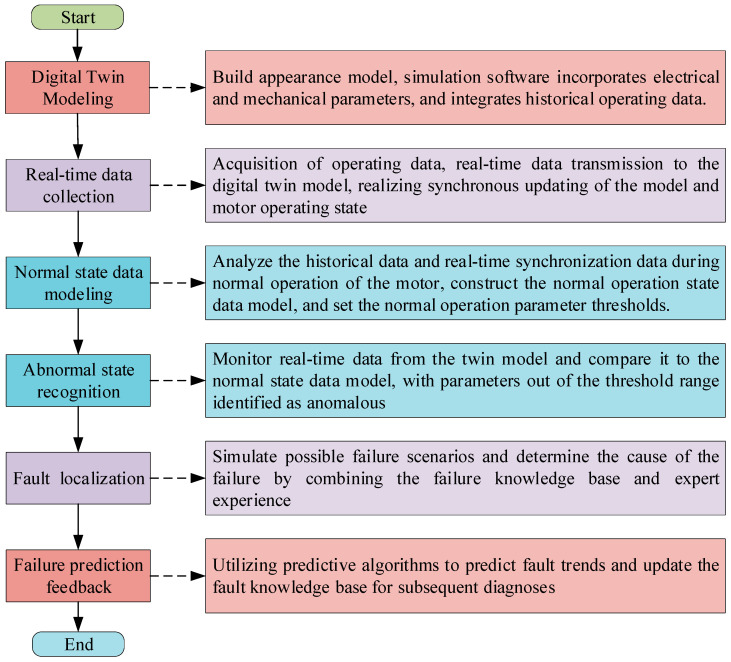
Digital twin-based motor fault diagnosis flow.

Digital twin technology, as an emerging digitalization tool, has brought a new light to the field of motor troubleshooting. Digital twins play a key role in system safety and risk assessment, which are closely related to worker safety and security. When heavy machinery fails, the consequences are unimaginable. In the field of electrical machinery, electrical and mechanical failures can lead to abnormal operation of the equipment, which not only interrupts production and causes material losses, but also jeopardizes the lives of workers. Digital twin technology can build a virtual model to map the operating status of equipment in real time. With the help of multi-source data acquisition and analysis technology, it digs deep into the equipment operation data, finding potential faults and warnings in real-time, so that workers can take precautions in advance to avoid safety accidents [77]. Figure 6 shows the flow chart of motor fault diagnosis based on digital twins.

### 4.1. Key Techniques for Fault Diagnosis of Digital Twin Motors

#### 4.1.1. Modeling Techniques for Multi-Source Data Acquisition

Data are the foundation of digital twins; accurate and comprehensive data acquisition and processing are the key to realizing motor fault diagnosis. Sensor networks and remote monitoring systems are important means of data acquisition and can obtain multi-source data such as vibration, temperature, current, voltage, etc., of motos. Reference [78] proposed a blockchain-based data management method that can effectively solve the problems of data storage, access, sharing and authenticity, and provide stable and reliable data support for digital twin motor fault diagnosis. In [79], multiple sensors are utilized to collect the operational data of wind turbines to provide support for subsequent analysis. The raw data collected often have problems such as noise and missing values, which need to be cleaned, integrated and formatted. Reference [80] collected data from three-axis acceleration sensors in a light truck transmission test bed, and constructed a virtual model containing a regular model, a geometric model and a dynamic model to provide data support for subsequent analysis. Reference [81] proposed a digital twin method for motor fault diagnosis based on variational modal decomposition (VMD) and dynamic vibration modeling. The experimental bench was equipped with two acceleration sensors, and the acquisition card recorded the vibration signals for 30 s at a sampling frequency of 125 kHz, and the test was carried out at a speed of 1200 rpm and a load of 11 Nm. The accuracy and reliability of the gearbox fault diagnosis were improved by setting different faults on the gearbox and obtaining the vibration signals under different states.

#### 4.1.2. Digital Modeling Techniques

Digital modeling is one of the core techniques of digital twins and is used to construct a virtual model of a motor to simulate its physical behavior and performance. Commonly used modeling tools include Unity3D, ANSYS, MATLAB (Simulink), COMSOL Multiphysics, etc. [82]. The multiscale modeling approach based on standardized model segmentation and assembly in [78] improved the versatility and adaptability of the generated model. Reference [79] used COMSOL Multiphysics software to establish a digital twin model of an induction motor, and realized high-precision simulation of the motor operation state by precisely setting the model parameters and boundary conditions. Reference [83] proposed a digital twin model constructed based on the double-pulse characteristic and impulse excitation of a single-degree-of-freedom nonlinear system. By setting parameters such as the attenuation coefficient and high-frequency resonance frequency, bearing signals with different fault sizes could be generated to improve the computational efficiency. Reference [84] proposed a fault diagnosis method for electric machines based on a digital twin and spatio-temporal graph convolutional network, using ADMAS software to construct a dynamic model of rotating mechanical devices, simulating the contact pairs between components through a contact force model, and verifying the consistency between the virtual model and the physical entity using the cosine similarity. Reference [85] explored induction motor fault diagnosis using the three-dimensional finite element method (FEM) to construct an induction motor model, taking into account the non-linear BH characteristics of the motor, the skin effect, the changes in material properties with temperature and other factors, so as to make the model as close as possible to the actual motor.

#### 4.1.3. Deep Data Analysis Mining Techniques

Data analysis and mining technology is an important support for fault diagnosis of digital twin motors and is used to analyze the collected data and extract fault characteristics to achieve accurate diagnosis and prediction of faults. Statistical methods, machine learning and deep learning algorithms are widely used in this field [86]. Reference [87] used deep learning algorithms to analyze motor current signals and automatically extract fault features to achieve effective diagnosis of motor faults. Reference [88] proposed a data-driven digital twin system based on the integration of virtual modeling, process monitoring, diagnosis and optimal control to achieve comprehensive management and fault diagnosis of the motor operation process. Reference [89] calculated the average integrated power spectral density (AIPSD) of vibration signals and extracted the fault characteristic frequency bands by analyzing the AIPSD values of different frequency bands. The AIPSD values were analyzed using one-class support vector machine (OCSVM) to determine the fault onset time and realize early fault detection. Reference [90] utilized machine learning classification algorithms for fault diagnosis. Algorithms such as Linear Discriminant and Support Vector Machines were selected after detecting deviations in the values of the dynamic model parameters, with a test accuracy of 80%. Reference [91] proposed a physically driven cross-domain digital twin-based framework that utilized the sequential frequency cyclic spectrum (CSC) correlation method to process the signal, improving the model’s fault diagnosis accuracy under non-stationary operating conditions through adversarial training with the double Fourier transform and gradient reversal layer (GRL).

#### 4.1.4. Multimodal Visual Interaction Technology

Visualization technology can present simulation results and data analysis resulting from digital twin models to users in an intuitive way, making it easy for the user to understand results and make decisions. Such technology relates to multiple aspects, such as data acquisition, modeling, integration, processing, display and interaction. The visualization of a digital twin model using Unity 3D mentioned in [78] can intuitively display the operating status and fault information of the motor, and the user can also gain insight into the internal structure and operating details of the motor through interactive operations. Reference [92] presents data features in a visual way by showing STFT matrix analysis plots, improved RGB acoustic images and their thermograms to help understand the fault diagnosis process and results. Reference [93] proposes a high-fidelity multi-physics field model for generating permanent magnet synchronous motor (PMSM) fault data, using waterfall plots, spectrograms, wavelet scale plots and envelope spectra to visualize vibration data when analyzing the vibration response, helping to observe and analyze vibration characteristics, embodying the auxiliary role of visual interactions for data analysis.

#### 4.1.5. Real-Time Update Synchronization Technology

The real-time update and synchronization technique of digital twins is a technical system that integrates the techniques of data acquisition, data transmission, processing and analysis of raw data, as well as model updates based on mapping relationships and time synchronization, to achieve real-time consistency between the state of the digital model and the physical entity. Reference [94] collects real-time image streams of physical wind turbines by UAVs and transmits them to the cloud, where they are preprocessed and fed into the edge client; subsequently, the deployed RDSS-YOLO neural network performs real-time detection and semantic segmentation. The edge client uploads the analysis results to the cloud server, realizing the real-time mapping of the physical wind turbine state in the virtual space. Reference [95] proposed a digital twin monitoring system for induction motors based on IoT sensors and thermal–magnetic finite element analysis, sending the fault data collected by the sensors to a cloud platform in real time via an ESP32 microcontroller to realize real-time updating and synchronization of the digital model with the data of physical entities. Reference [96] proposed a permanent magnet synchronous motor fault diagnosis method for a coal mine belt conveyor based on a digital twin and an improved sparrow search algorithm-optimized random forest (ISSA-RF) method, realizing interactive data transmission between the physical entity and the digital twin model through communication protocols, such as OPC UA, to reflect the motor’s operation status and real-time fault diagnosis and monitoring in real time.

### 4.2. Application of Digital Twins in Motor Fault Diagnosis

**Figure 7:**
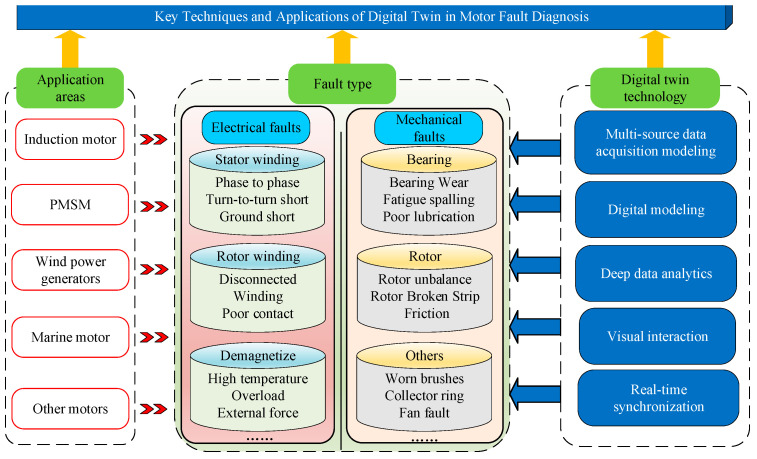
Application of digital twins in motor fault diagnosis.

Digital twin technology has shown excellent application value in electric motor fault diagnosis. According to literature statistics, in the field of motor fault diagnosis, digital twin technology is mainly applied to two major types of induction motors and permanent magnet synchronous motors, and the technology is also involved in scenarios such as wind power generation, ship propulsion, electric vehicles and aviation motors. In this section, the digital twin motor fault diagnosis technique is discussed in terms of these two motor types and different application scenarios. Figure 7 shows a break-down of the application of digital twins in motor fault diagnosis.

#### 4.2.1. Induction Motor Troubleshooting

Induction motors are widely used in industry, household appliances and transportation, mainly for driving fans, pumps, compressors and other equipment, and accurate fault diagnosis is crucial. Reference [97] establishes a digital twin model based on multi-physics field simulation for induction motors, expands the label-scarce dataset by generating simulated data under different fault states and realizes the transfer of knowledge from the virtual space to the physical space by using deep domain adaptation, which effectively improves the accuracy of fault diagnosis. Reference [98] proposed an induction motor fault detection method based on deep learning and a digital twin that utilizes the digital twin model to monitor the motor operation state in real time and analyzes the monitoring data by bringing in a deep learning algorithm that can accurately identify multiple fault types. Reference [99] proposed a digital twin service unit for detecting AC motor stator turn-to-turn short circuit faults, providing an effective means for motor maintenance using real-time measurements and Linux Robotics Operating System (ROS) simulation of motor behavior to achieve fault warning. Reference [100] established a digital twin model of an induction motor through the finite element method to study faults such as rotor broken bars and analyzed changes in the electromagnetic characteristics of the motor during faults, providing a strong basis for fault diagnosis. Reference [101] constructed a digital twin architecture named “Ramanujan DT” for monitoring and detecting periodic faults of motors and experimentally verified the effectiveness and robustness of the model in recognizing common faults (such as broken bars and stator winding faults) under a variety of operating conditions and noise levels.

#### 4.2.2. Troubleshooting Permanent Magnet Synchronous Motor

Permanent magnet synchronous motors, with their high efficiency, high power density and excellent speed regulation performance, are widely used in the fields of new energy, the automobile industry and aerospace, and their fault diagnosis is crucial, for which digital twin technology provides an effective approach. Reference [102] proposed a PMSM fault diagnosis method based on digital twin modeling, through the establishment of an accurate digital twin model and real-time monitoring and simulation analysis of the motor operating state; the method proved able to detect faults in an appropriate amount of time and determine the types of faults. Reference [103] proposed a diagnosis method based on a data-driven digital twin model for early turn-to-turn short circuit faults in PMSM, realizing the accurate diagnosis of early faults by analyzing information such as the three-phase current residuals of the motor and combining the data with a non-linear autoregressive network to construct a digital twin model in the healthy state. Reference [104] proposed a data-driven reliability design optimization (RBDO)-based approach for sensor layout and fault diagnosis of PMSM that uses digital twin technology to construct an FEA model of the motor, simulate fault conditions and generate data.

#### 4.2.3. Troubleshooting Wind Turbines

Wind turbines work in harsh environments and have a high failure rate. Digital twin technology can realize real-time monitoring and fault diagnosis of wind turbine generators, and improve their operational reliability and maintenance efficiency. Reference [105] proposes a fault diagnosis method for a wind turbine drive train based on digital twin technology, which can accurately identify fault types with a diagnostic accuracy as high as 99.1% by establishing a digital twin model and incorporating real-time monitoring and simulation of the drive train’s operating status combined with an improved variational modal decomposition and particle swarm optimization least squares support vector machine algorithm. Reference [106] focuses on the field of wind turbine planetary gear fault diagnosis, using digital twin technology to build a digital twin model of planetary gears, combined with the EMD—ASO—SVM fault diagnosis method; the model can accurately and in a timely manner determine the health status of the planetary gears, which effectively solves the problems of traditional diagnostic methods such as the poor timeliness of data transmission, the weak effect of the visualization of the state monitoring and the untimely feedback of fault information. It can effectively solve the problems of poor data transmission, weak visualization effect of condition monitoring and untimely feedback of fault information of traditional diagnosis methods.

#### 4.2.4. Troubleshooting in Other Motor Fields

Digital twin technology provides a new way forward for ship motor fault diagnosis. Reference [107] constructed a fault diagnosis model of an unmanned ship integrated power system based on a digital twin system using a rule-based discriminative method, which could effectively identify the system fault and its type without the need for historical data and a specific physical model. Reference [108] utilized digital twin technology for fault diagnosis of electric motors in ship propulsion systems, and through real-time monitoring of the motor’s operating data and comparative analysis with the digital twin model, it proved able to detect faults and provide early warning in an appropriate amount of time to ensure the safe operation of the ship. In the field of aviation motors, ref. [109] used digital model simulation to obtain data, study the change in parameter characteristics of motors in normal and fault states, train and compare the fault identification ability of different machine learning models, and provide an effective method for the fault diagnosis of aircraft electric brake systems. In the field of fault diagnosis of various types of motor bearings, ref. [110] proposed a digital twin-driven discriminative graph learning network (DT-DGLN) for bearing fault diagnosis, which simulates the operating state and fault scenarios of the bearings by constructing a high-precision digital twin model and combining it with the domain-adaptation framework based on the graph learning strategy, migrating the knowledge of the simulated signals to the real signals and realizing cross-domain fault diagnosis. Reference [111] proposes a class incremental learning method MFIF-CIL that incorporates multi-fidelity information for digital twin-driven fault diagnosis of electric motor bearings, which combines the physical system, measurement data, and cosine similarity updating techniques to create a real-time digital twin platform of the axial system by using ANSYS, Inventor, 3DMax, and Unity3D, to achieve the real-time monitoring and simulation. In the field of electric vehicles, ref. [112] analyzes the vibration and current data during motor operation by constructing a digital twin model and combining advanced Transformer and convolutional layer techniques to achieve effective diagnosis of motor faults, which provides a strong support for preventive maintenance of motors.

#### 4.2.5. Comparative Analysis of Different Application Areas

Digital twin technology has significant advantages and distinctive features in multiple fields of motor applications. Induction motors are widely used in medical, machine tool and rail transportation fields, and digital twins and other technologies are used to improve the accuracy and timeliness of fault diagnosis. However, in industrial environments, the operating characteristics of induction motors are affected by load variations, temperature fluctuations and other factors, which makes it challenging to obtain accurate speed, torque and power data from digital twins; in the fields of electric vehicles, aerospace and robotics, the focus is on permanent magnet synchronous motors, with an emphasis on total lifecycle management to ensure efficient operation. In electric vehicles, aerospace and robotics, which focus on permanent magnet synchronous motors and full lifecycle management to ensure efficient operation, the digital twin model needs to consider the effects of load changes and the simultaneous occurrence of multiple faults such as turn-to-turn short circuits and rotor failures on the performance of the motors, which may increase the complexity of the model and the amount of computation, and it is difficult to completely accurately simulate the changes of speed, torque and power in different operating conditions; in new energy, ship transportation and other harsh environments, the use of digital twins, combined with algorithms to improve diagnostic accuracy and reduce fault losses, changes in environmental factors, such as wind speed and direction, and load changes, leads to changes in sailing state factors on the motor speed, torque and power, resulting in higher requirements—the digital twin model needs to be able to accurately simulate the impact of such load changes on the performance of the motor. Table 2 shows methodological improvements in motor fault diagnosis techniques based on digital twin technology compared to traditional methods.

Compared with traditional methods, digital twin technology in the field of motor fault diagnosis accuracy, timeliness and intelligence has significantly improved in different application areas, though it still has certain limitations. Table 3 shows a comparison of motor fault diagnosis methods based on digital twins.

## 5. Conceptualization of Digital Twin Applications in Fault Diagnosis of Three-Phase Synchronous Generators

Digital twin motor fault diagnosis technology is mainly applied to two major types of induction motors and permanent magnet synchronous motors, and the technology is also involved in wind power generation, ship propulsion, electric vehicles and aviation motors.

Multi-synchronous generators have been playing an increasingly important role in shipboard integrated power systems, and compared with conventional applications, generators differ greatly in their application environment, excitation system configuration, heat dissipation method and control method, which affects the overall life and reliability of generators. In this system, stator winding turn-to-turn short circuit faults cannot be ignored, but obtaining enough labeled data to train the model is both expensive and laborious; the question of how to effectively utilize multi-phase current signals is a challenging task. In this section, a data-driven and enhanced semi-supervised framework-based fault diagnosis method is proposed based on the digital twin technique, which presents a conceptualization of a multi-synchronous generator stator winding turn-to-turn short-circuit fault diagnosis method.

### 5.1. Data-Driven Digital Twin-Based Model Construction

**Figure 8:**
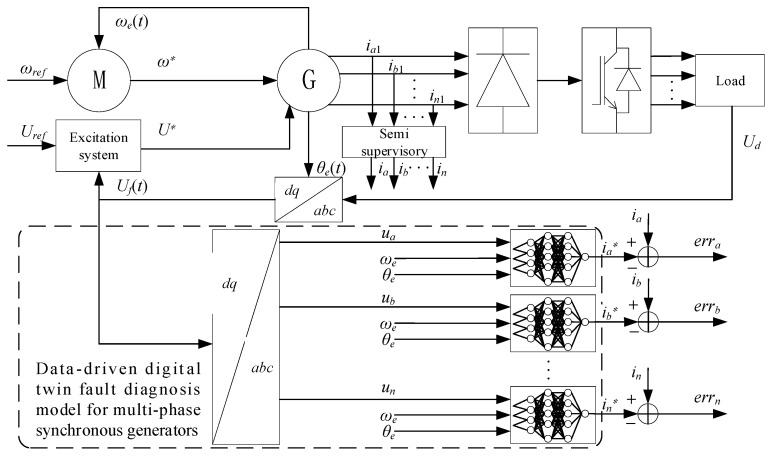
Data-driven fault diagnosis model for digital twin generators.

With the increasing size and complexity of power systems, the reliable operation of generators is essential to ensure the stability of power supply. Data-driven technology realizes effective diagnosis of motor faults with the help of a large number of data and advanced algorithms. In this paper, a data-driven digital twin-based generator fault diagnosis model is proposed to realize accurate diagnosis and prediction of generator faults by constructing a digital twin that is highly similar to the actual generator, and collecting and analyzing operation data in real time. The model utilizes deep learning and machine learning algorithms to learn from a large number of historical data and automatically extract fault features, improving the accuracy and efficiency of fault diagnosis. The data-driven digital twin generator fault diagnosis model is shown in Figure 8.

#### 5.1.1. Defining the Model Architecture

First, n-parallel non-linear autoregressive exogenous (NARX) networks were chosen to build the digital twin model. n NARX networks have the ability to handle dynamic systems and capture complex nonlinear relationships between inputs and outputs, which is crucial for simulating the operating characteristics of permanent magnet synchronous motors. The n-parallel networks correspond to the n-phase currents of the motors, which allow for the accurate simulation of the currents of each phase independently and collaboratively.

#### 5.1.2. Data Acquisition

For multi-synchronous generators, a variety of key parameter data are collected in the healthy operating state. They mainly include the three-phase voltage signals $u_{a},u_{b}$, …, $u_{n}$, which reflect the input excitation of the motor; the electrical angular velocity, which reflects the rotational speed of the generator $\omega_{e}$; and the electrical angle $\theta_{e}$, which is used to determine the rotor position of the motor. In addition, the actual three-phase currents $i_{a1},i_{b1}$, …, $i_{n1}$ of the generator are also recorded as important reference data for subsequent model training and validation. To ensure the comprehensiveness and reliability of the collected data, data collection is performed under different operating conditions, covering a variety of rotational speeds and load conditions. Different reference speeds and reference excitation input voltages are set, and the generator is allowed to run stably under different load torques, while the data on the above parameters are continuously collected at an appropriate sampling frequency to ensure that the collected data can fully reflect the various operating characteristics of the motor in a healthy state.

#### 5.1.3. Model Training

First, divide the dataset. The preprocessed health data are divided into a training set, validation set and test set. Generally speaking, the training set is used for learning the parameters of the model, the validation set is used to adjust the hyperparameters such as the number of layers and neurons of the model network during the training process to prevent the model from overfitting, and the test set is used to evaluate the generalization ability of the trained model on unseen data. The division ratio is 70% for the training set, 15% for the validation set and 15% for the test set. Next, we have the input and output settings. The preprocessed multi-phase voltages, electrical angular velocities and electrical angles are used as inputs to the n-parallel NARX networks, and the corresponding three-phase currents $i_{a}^{*},i_{b}^{*}$, …, $i_{n}^{*}$ are used as the desired outputs of the networks. Finally, the n-parallel NARX networks are iteratively trained using the training set data. During the training process, the error between the predicted output and the desired output of the model is calculated by the back-propagation algorithm, and the weights and bias parameters of the network are adjusted according to the error, so that the prediction results of the model gradually approximate the actual generator current response.

### 5.2. Sample Generation Method Based on Semi-Supervised Learning Framework

**Figure 9:**
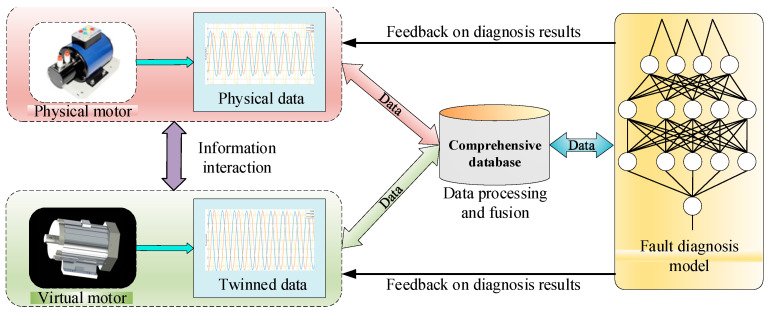
Digital twin motor fault diagnosis architecture.

In the field of three-phase synchronous generator fault diagnosis based on digital twins, a sample generation method based on a semi-supervised learning framework is extremely valuable. The method comprehensively collects generator operation data with the help of digital twin technology, on the basis of which a small number of labeled fault samples and a large number of unlabeled samples are integrated to carry out the work. On the one hand, unsupervised learning tools such as clustering and dimensionality reduction are used to mine the potential patterns of the unlabeled data, and initially generate valuable sample features; on the other hand, active learning and collaborative training strategies are combined to select key samples from the unlabeled data for labeling and allow multiple models to collaborate with each other in order to expand the set of labeled samples, to ultimately generate rich and high-quality samples for accurate and efficient fault diagnosis model training. Figure 9 shows the architecture of digital twin motor fault diagnosis.

#### 5.2.1. Background on the Selection of Semi-Supervised Clustering Algorithms

In the data collection process of generator fault diagnosis, it is often difficult to obtain a large number of fault data with accurate labels, due to the episodic nature of faults and the high cost of accurately labeling faults, which requires specialized knowledge and practical experience. However, data from normal operating conditions are relatively easy to collect in large quantities. In this case of uneven data distribution, traditional supervised learning methods are difficult to perform ideally due to their reliance on a large number of labeled data for training. And although unsupervised learning methods can handle unlabeled data, they lack explicit guidance on specific failure modes, and the diagnostic accuracy is also affected. Therefore, semi-supervised learning algorithms emerge, which combine the advantages of supervised learning and unsupervised learning, and can utilize a small number of labeled fault data and a large number of unlabeled normal operation data for model training to achieve more accurate fault diagnosis under limited data resources.

#### 5.2.2. Initial Classifier Training

A small number of fault data that have been manually labeled are used to train an initial classifier, and these labeled data are like “seeds” that provide the classifier with the very first learning samples to give it an initial understanding of the characteristics and patterns of the fault data. In three-phase synchronous generator fault diagnosis, these labeled data contain information about various operating parameters such as current, voltage, vibration frequency, etc., which has have been previously recorded at the time of a stator winding short circuit fault occurrence. Using common machine learning models such as support vector machines, decision trees or neural networks as classifiers, the classifiers establish preliminary fault judgment rules by learning from these labeled data.

#### 5.2.3. Unlabeled Data Forecasting

When the initial classifier is trained, it is used to make predictions on a large number of unlabeled data. These unlabeled data are mainly data collected during the normal operating state of the generator, but may also contain data from some unknown state. The classifier will judge each unlabeled sample based on what it has learned on the labeled data, giving the probability that it belongs to a certain fault type or normal state.

#### 5.2.4. High-Confidence Sample Screening and Addition

After making predictions on unlabeled data, it is necessary to filter out samples with high prediction confidence. Confidence reflects the degree of certainty of the classifier about its own prediction results, and when the probability of a sample being predicted for a certain category exceeds a certain threshold, the prediction is considered to be more reliable. These high-confidence samples are selected from the unlabeled dataset and added to the labeled dataset.

#### 5.2.5. Model Retraining and Iteration

The labeled dataset is expanded by adding high-confidence samples, and the classifier is retrained using the updated labeled dataset. During this retraining process, the classifier is able to learn more information about the samples, including those that were originally unlabeled but are now given reliable labels. With one iteration, the classifier continuously adjusts its parameters and judgment rules, gradually adapting to more different data features and becoming more capable of distinguishing between faulty and normal states, thus achieving model optimization. In this section, a large number of labeled samples with high confidence, $i_{a},i_{b}$, …, $i_{n}$, are screened.

By using a semi-supervised clustering algorithm with a self-training algorithm, combined with a small number of labeled fault data and a large number of unlabeled normal operation data for training, the semi-supervised learning model is able to fully explore the potential information in the data with limited data; combined with the data-driven generator digital twin model, the method can improve the efficiency and accuracy of fault diagnosis for the multi-phase synchronous generator and provide a powerful guarantee of its reliable operation.

## 6. Conclusions

Digital twin technology, as an emerging digital approach, has been widely studied and applied in the field of motor fault diagnosis. This paper explores the application of digital twins in motor fault diagnosis, comprehensively describing its development status, technical principles and application examples, while providing in-depth analysis of the challenges faced and looking forward to future development directions. This paper draws the following detailed conclusions.

### 6.1. Technological Advantages and Application Achievements

(1) Realization of real-time accurate monitoring and diagnosis: Digital twin technology, with the help of multi-source data acquisition modeling, digital modeling, in-depth data analysis and mining, as well as multi-modal visual interaction, real-time updating and synchronization and other key technologies, enables building virtual models that can provide highly accurate simulations of physical entities. Motor operation data are collected in real time through sensors, processed and analyzed to drive virtual models, realizing real-time mapping and accurate simulation of the motor operation state. This makes it possible to capture subtle changes in motor operation over time, accurately determine the type, location and severity of faults, and also predict the development trends in faults based on historical data and models, providing a basis for early intervention and maintenance.
(2) Multi-field application with outstanding results: In the field of induction motor fault diagnosis, the digital twin model is based on multi-physical field simulation, which can expand relevant datasets and provide a strong basis for diagnosis by simulating the changes in the electromagnetic characteristics of motors during faults. In the field of permanent magnet synchronous motors, an accurate digital twin model can monitor and simulate and analyze the running status of the motor in real time, discover early faults in an appropriate amount of time, and ensure efficient and stable operation. Wind turbines work in harsh environments; digital twin technology combined with related algorithms can increase diagnosis accuracy up to 99.1%, effectively solving the many problems of traditional diagnostic methods. In other motor applications, digital twins provide a new way to diagnose faults in complex power systems and motor equipment, and provide effective support for fault diagnosis and preventive maintenance of related equipment.

### 6.2. Challenges to Be Urgently Addressed

(1) The existing literature refers to the following insufficient aspects: Firstly, the lack of standardization and specification limits development. There is a lack of unified technical standards and specifications, and different studies and applications differ greatly in data formats, interface standards, modeling methods, diagnostic processes, etc., which makes it difficult for systems to be compatible and work together. This not only increases the cost and difficulty of technology promotion, but also hinders cross-domain cooperation and technological innovation, limiting the wide application and in-depth development of digital twin technology in the field of motor fault diagnosis. Secondly, the elaboration of cross-domain application is not in-depth. For the cross-domain application of motor fault diagnosis methods in different fields, the literature is not deep enough. Although applications in industrial manufacturing, energy, aerospace and other fields are mentioned, there is a lack of detailed description on how to make targeted adjustments and optimizations according to the characteristics and needs of different fields.
(2) Limitations of existing methods: First, there are data-related problems. There are problems with data acquisition and quality, and it is more difficult to acquire accurate, complete and high-quality data in complex environments. The problem of data imbalance is prominent, with more normal state data and less fault state data, leading to a decrease in the diagnostic ability of models for faults. The second is the problem of model accuracy and real-time performance. Model accuracy is constrained by the quality and quantity of data as well as the model design and training process. In practical applications, complex working conditions and variable environments make it difficult for the model to accurately reflect the real state of the motor. Third, there is the problem of sustainable diagnosis and generalization ability. Existing diagnostic models have difficulty maintaining accuracy and reliability in the face of equipment aging, maintenance activities and emerging failure modes.
(3) The main challenges of integrating DTs for fault diagnosis are the following: Firstly, there is the challenge of data processing and transmission. Data processing capabilities are highly required to deal with large-scale, multi-source, heterogeneous data, and motor fault diagnosis requires the integration of sensor data, historical data, maintenance records and other data in various formats and sources, which makes all these data difficult to process. Second, modeling challenges deserve mention. Especially for complex electromechanical equipment, it is difficult to accurately model the interactions between components, which affects the accuracy and reliability of the model. Third, there are security and privacy challenges. Data security and privacy protection are crucial. DT systems involve a large amount of sensitive data and, the openness and interconnectivity of these systems increase security risks. The question of how to realize data sharing and effective utilization under the premise of data security is a major challenge.

### 6.3. Clear Direction for Future Development

(1) Efforts are being made to solve existing problems: In data processing and transmission, advanced algorithms are being developed to remove noise, fill in missing values and improve data quality; the integration of big data elements related to data transmission with AI technology is significant, and needs to be analyzed and reviewed in terms of the reliability of data transmission, the adaptability of AI algorithms, the validity of the fusion of data and algorithms, and the security and privacy protection of relevant methods. AS for model optimization, new model structures and algorithms are being explored, such as lightweight neural networks and distributed computing models, to improve computational efficiency and model interaction in real time while ensuring accuracy. These optimizations should be based on existing digitalization and information technology and other related industry standards, such as GB/T 23011—2022 “Informationization and Industrialization Convergence Digital Transformation Value Benefit Reference Model” [113] and GB/T 28827.1—2022 “Information Technology Services Operation and Maintenance” [114], to strengthen the cooperation between industry, academia and research institutes, to conduct in-depth research on the theory of digital twins, to provide feedback from industry about practical application problems, and to integrate resources in research institutes. It is important to overcome key issues such as data formats, interface standards and modeling methods, among other issues, as well as to meet the requirements of practical applications and help create digital twin industry standards. It is also important to deepen the integration of multiple disciplines, promote the intersection of computer science, mathematics, physics and other disciplines, introduce cutting-edge theories and methods to optimize digital twin models, improve their performance, and provide technical support for motor fault diagnosis.
(2) Strengthening multidisciplinary integration and innovation: We should continue to deepen the cross-integration of computer science, mathematics, physics and other multidisciplinary disciplines, and draw on the cutting-edge theories and methods of each discipline. We must also combine new advances in artificial intelligence to develop smarter and more efficient digital twin models; use mathematical optimization algorithms to improve the processes of model training and parameter adjustment; establish more accurate physical models of electric motors based on the principles of physics, so as to improve the performance and adaptability of the digital twin models; reduce human intervention and direct contact; and carry out risk assessments to ensure the safety of personnel and reduce material losses.

## Figures and Tables

**Table 2:** Improvement of motor fault diagnosis method based on digital twins.

Type of Motor	Diagnosis Method	Inadequacy of Traditional Methods	Digital Twin Improvements	Reference
PMSM	Based on search coils	Complex hardware Poor generalizability High interference impact	Highly adaptable and versatile Intelligent calibration and self-updating High fault extraction capability	[35,96]
Induction motor	Based on data-driven and physical models	Relies on large amounts of failure history data Rely on accurate physical models Rely on human guarding	Digital twin system simulates normal operating values Enables intelligent monitoring and optimization	[41,97]
Wind turbine	Model-based and data acquisition monitoring system	Reliance on expert experience High cost of data acquisition Insufficient precision	Analyze failure warning using historical operating condition information Early detection of potential faults using residual values as fault characterization variables	[59,109]
Marine propulsion motor	Based on stacked self-encoders and convolutional neural networks	Effectively detects early failures and determines when they occur	Digital twin model expansion for scarce datasets Strong adaptability and generalization to non-linear fault data	[61,107]
Other motors	Fault frequency-based approach	Susceptible to noise interference Difficult to acquire data	Good data quality, accuracy and reliability Strong model generalization ability	[62,95]

**Table 3:** Comparison of digital twin based motor fault diagnosis methods.

Type of Fault	Diagnosis Method	Monitoring Indicators	Data Supports	Strengths and Limitations	Reference
Gear contact fatigue damage	VBDM-DT model	Model input torque	The model error is 8.34%	Intelligent calibration for high model fidelity; Model functionality and applicability need to be expanded	[80]
Motor inner ring failure, outer ring failure	Model based on time series fusion Transformer	Diagnostic Precision, Accuracy, Recall, F1	Accuracy 99.99%, Recall 99.98%, F1 Score 0.99	Excellent performance in overall performance indicators; Generalization ability in real scenarios needs to be strengthened	[81]
Bearing failures	Parameterized CNN	Vibration response characteristic for frequency and amplitude	Diagnostic accuracy 94.0741%	Diagnosis without fault data, high diagnostic accuracy; Need to improve the digital twin model	[83]
Demagnetization faults, dynamic eccentricity faults	SFT, CNN	Fault state	Accuracy 99.72%, 10 times more efficient	High fault recognition accuracy and stability; Unexpanded fault diagnosis types	[89]
Motor surface damage	RDSS-YOLO neural network	Average Precision, Recall	Average Precision of 95.7%, Recall rate 96.8%	Higher detection accuracy; Unable to detect internal component damage	[91]
Bearing wear, corrosion	Multi-objective particle swarm optimization algorithm	Mean absolute error	RMSE is 0.051	Reduced dependence on actual data, high diagnostic accuracy; More modes under actual working conditions are not considered	[110]
PMSM turn-to-turn short circuit faults	NARX Network	Three-phase current residuals, relative mean square error	\	Reduced dependence on actual data, high diagnostic accuracy; More modes under actual working conditions are not considered	[96]
Rotor broken bars, stator winding failure, rotor misalignment	PCCDP, clustering-based metric learning	Diagnostic accuracy	High diagnostic accuracy in case of label scarcity	No need for fault data and precise motor parameters; Separate digital twins are required for different motor models	[97]
Failure of rotor bar breakage	MCSA	Low-sideband harmonic components	Experimental LSH amplitude increased by 3.62 dB when the bar was broken, and by 3.34 dB in the simulation	Simple model construction, good agreement between simulation and experimental data; Variation in motor parameters with temperature, frequency and saturation are not considered	[98]
Axis misalignment	ResNet-50 deep learning	Model testing accuracy, validation accuracy	Diagnostic accuracy of 98.6%	AI applications to improve diagnostic accuracy and efficiency; Difficult and costly to obtain sufficient high-quality learning data	[112]
Motor inner ring failure, outer ring failure	Inverse PINN	Vibration signal spectrum	Diagnostic accuracy of 98% on average	Effectively addressing sample imbalances; Need to further improve the quality and diversity of generated data	[77]
Short circuit failure	Artificial intelligence-based methods, signal processing and feature extraction techniques	Current, signal reflection parameters	Diagnostic accuracy of 98.6%	Artificial intelligence applications to improve diagnostic accuracy and efficiency; Difficult and costly to obtain sufficient high-quality learning data	[84]
Motor bearing outer ring spalling	High-fidelity multi-physics field finite element modeling	Vibration response, torque	Tolerance of about 3%	Solves the problem of data scarcity; Models bias in predicting certain vibrations	[90]
Collector ring oxidation	FEM, thermomagnetic intensity coupling simulation and analysis	Motor phase current, case temperature	Current measurement error 4.4%, temperature measurement error 10%	Non-intrusive monitoring with low errors; No mention of the stability of the system operating under complex conditions	[92]
Turn-to-turn short circuit	Digital twin-based and improved Sparrow Search Algorithm	Current, voltage, speed	Diagnostic accuracy 98.2143%	High diagnostic accuracy due to 3D visualization and monitoring; Aability to apply theory to practice needs to be improved	[93]
Rotor breaks	FEA, Fuzzy Logic Control	Motor losses, temperature, efficiency	Rapidity varies across models	Circuit modeling is simple and efficient; Limited accuracy when dealing with complex situations	[94]
Rotor breaks	Digital shadowing system based on efficiency modeling	Machine loss, efficiency	Diagnostic accuracy 99.99%, F1 Score 0.99	Good adaptability to different fault severities; Range of applications to be expanded	[95]
Rotor broken strip, broken end ring	Multi-cage fault analysis model of electric machine based on winding tensor method	Characteristic fault harmonic components	Good agreement between simulated and experimental data	Fast calculation speed and simple calculation of model parameters; Variation in motor parameters with temperature is not considered	[99]
Rotor imbalance, broken bars	FEM	Stator and rotor current, coil voltage, electromagnetic torque	Measured stator current at 60 Hz 5.780 A, modeled value 5.865 A	Accurate and efficient modeling of difficult-to-measure electromagnetic quantities; Does not take into account magnetic saturation	[100]
Broken bar failure, winding failure	RPT, Bayesian updating	Fault characteristic frequency	BRB faults can be effectively diagnosed at −10 dB noise level	Strong robustness to noise interference; Lacks context awareness	[101]
Rotor breaks	MCSA, FEM	Time–frequency-domain current signal characteristics	Average error 1.55%	Non-invasive testing with low diagnostic error; Three-dimensional FEM models are computationally complex	[103]
Inner ring, outer ring failure	IVMD combined with PSO-LSSVM	Time–frequency-domain characterization of vibration signals	Diagnostic accuracy 99.1%	High visualization and diagnostic accuracy; Experimentally unstudied more types of faults	[105]

## Data Availability

Not applicable.

## Abbreviations

The following abbreviations are used in this manuscript:

SVM	Support Vector Machine
KNN	K-Nearest Neighbor
CNN	Convolutional Neural Network
FFT	Fast Fourier Transform
GUI	Graphical User Interface
LSTM	Long Short-Term Memory
DRL	Deep Reinforcement Learning
ASO	Atomic Search Optimization
FEM	Finite Element Method
FT	Fourier Transform
DWT	Discrete Wavelet Transform
VMD	Variational Mode Decomposition
PCCDP	Percentage Contrast Current Dot Pattern
NARX	Nonlinear Autoregressive with Exogenous Inputs
MCSA	Motor Current Signature Analysis
STFT	Short-Time Fourier Transform
GCN	Graph Convolutional Network
